# Public Health Financing and Responses to COVID-19: Lessons from South Korea

**DOI:** 10.3390/healthcare10040750

**Published:** 2022-04-18

**Authors:** Hyeki Park, Boram Sim, Bo Zhao, Eun Woo Nam

**Affiliations:** 1 HIRA Research Institute, Health Insurance Review and Assessment Service (HIRA), 60, Hyeoksin-ro, Wonju-si 26465, Korea; hyekipark@gmail.com (H.P.); simbr12@hira.or.kr (B.S.); 2 Department of Public Health, Graduate School, College of Medicine, Yonsei University, 50 Yonsei-ro, Seodaemun-gu, Seoul 03722, Korea; 3 Department of Health Administration, Graduate School, Yonsei University, 1 Yonseidae-gil, Wonju-si 26493, Korea; zhaobo@yonsei.ac.kr; 4 Yonsei Global Health Center, Yonsei University, 1 Yonseidae-gil, Wonju-si 26493, Korea

**Keywords:** Korea, COVID-19 response, health financing, health insurance, health policy

## Abstract

Health financing strategies contribute significantly to containing the outbreak of the Coronavirus disease 2019 (COVID-19). This study aims to reassess Korea’s financing strategies in response to COVID-19 in 2020, to ascertain its effects and sustainability. The Joint External Evaluation tool was adopted to analyze the data collected from government reports, official statistics, and other sources. Findings show that Korea could maintain a low incidence and fatality rate compared with other countries, at low costs. It was a result of rapidly procured healthcare resources based on laws and policies established after the 2015 epidemic, and the National Health Insurance. However, to achieve long-term sustainability, it is important to enhance the financial stability of the national health insurance and increase the proportion of the public sector in healthcare resources.

## 1. Introduction

After the first case of the Coronavirus disease 2019 (COVID-19) was reported in December 2019 in Wuhan, China, the disease spread rapidly worldwide within 2 to 3 months [1]. The World Health Organization (WHO) officially declared it a public health emergency of international concern (PHEIC) in January 2020 [2]. Accordingly, countries have taken urgent action to protect their populations from the COVID-19 pandemic and its social effects [3]. Governments have strived to increase their capacity to test, trace, and treat COVID-19 patients while maintaining essential health services [4]. Countries have transferred additional funds from government budgets to the health sector to mobilize resources and accelerate emergency spending [5]. However, to make health systems more resilient, health financing plays a crucial role in the government’s response to the pandemic [6]. 

Korea identified the first cases of COVID-19 on 20 January 2020, and subsequently experienced the first wave of the outbreak in mid-February, with a peak of 909 newly confirmed cases on 29 February 2020. The second and third waves of the pandemic continued from August to September and November to December 2020, respectively [7]. In response, the Korean government has set a strategy for massive testing, contact tracing, and treatment, referred to as the “3T approach” [8]. With this strategy, Korea flattened the COVID-19 curve three times, resulting in a relatively low incidence and fatality rate in 2020 [9]. Therefore, despite being a country neighboring China, Korea is not the most affected worldwide [10].

Accordingly, several studies have reviewed Korea’s strategy to respond to COVID-19, including health, economic, and social measures [10,11,12]; however, the underlying health financing mechanism and cost have not yet been investigated. Given that the pandemic is an ongoing global crisis, Korea and other countries must review the factors contributing to successful or failed responses to COVID-19. Therefore, this study aimed to explore the COVID-19 response strategies and related public health financing and budget mobilization undertaken during the pandemic in Korea. 

## 2. Materials and Methods

This study analyzed public health financing and COVID-19 responses in Korea in 2020, based on the Joint External Evaluation (JEE) tool developed by the WHO [13]. The tool was developed to assess countries’ ability to promptly and effectively prevent, detect, and respond to international public health crises [13]. Therefore, it is a suitable and internationally comparable tool for evaluating the response to COVID-19. We modified the JEE tool to consider the epidemiological characteristics of the pandemic and focused on financial strategy analysis. The preparedness section examines the background of the health financing system and infection prevention budget that existed before the prevalence of COVID-19. In the detect and response section, budget mobilization, allocations, and purchasing and financing mechanism adjustments for the COVID-19 response are included. Specifically, the detection section includes diagnosis and surveillance, and the response section includes the adjustment of financing mechanisms, including medical countermeasures. Additionally, we included the budget for government subsidies for the sustainability and resilience of health and social systems.

This study collected publicly available official reports, documents, and statistics from the Korean government, public agencies, and the national assemblies. We extensively reviewed the health financing policies implemented in 2020; discussions of the lessons learned from the first year of the pandemic in Korea; and the public health laws and policies, which were amended after the Middle East Respiratory Syndrome (MERS) epidemic in 2015. 

## 3. Results

### 3.1. Preparedness

#### 3.1.1. Background of Health Financing System in Korea

The Korean healthcare system is dependent on the private sector. Moreover, more than 90% of healthcare institutions operate privately [14]. However, through the mandatory designation system, all public and private medical institutions participate in the NHI [15]. Among the three dimensions of universal health coverage (UHC)—that is, population coverage, service availability, and financial protection—Korea has achieved a coverage rate of over 95% for the population under the NHI [16]. However, service availability and financial projection progress remain challenging [17]. Though the scope of NHI coverage has continued to expand, the coverage rate remains steady at a level of slightly over 60% [18]. 

Accordingly, the Korean government announced in 2019, the 2019–2023 Comprehensive Plan of National Health Insurance, and set the policy goal of the plan to operate a sustainable system while simultaneously expanding coverage [19]. The government has been trying to increase these rates by covering essential services that are not covered by insurance and setting an upper limit for copayments [20]. Meanwhile, it is trying to optimize healthcare use for increased financial sustainability by managing the increasing rates of outpatient clinic use, duration of hospital stay, and the management rate of non-essential expenses [21,22,23]. 

#### 3.1.2. Infectious Disease Control System and the Budget in Korea

After the MERS epidemic in 2015, Korea significantly amended related laws and established a systematic response system [24,25]. Accordingly, the Korea Disease Control and Prevention Agency (KCDA) was put in charge of responding to levels 1 and 2 of national crises, during outbreaks of infectious diseases. The Ministry of Health and Welfare (MoHW) participates in level 3, where community transmission begins; the Central Disaster and Safety Countermeasures Head Quarter (CDSCHQ) was established to function at level 4, the most severe level, at which all ministries jointly participate in responding to the crisis. At this time, the prime minister takes the position of the CDSCHQ and manages all responses to achieve unification. Costs for testing, quarantine, and treatment are paid for by the central and local governments and the National Health Insurance (NHI) through the Infectious Disease Control and Prevention Act. In January 2020 [26,27], before the COVID-19 pandemic, Korea’s budget for responding to infectious diseases was KRW 19.2 billion (USD 16.78 million).

### 3.2. Detect and Response

#### 3.2.1. Overall Government Budgeting during the COVID-19 Pandemic

The budgets for responding to COVID-19 were secured through the appropriation of funds for existing projects, disbursement of reserve funds, and supplementary budgeting. Initial responses were made using the regular budget allocated for infectious disease responses and funds from the NHI. Fund shortages were managed by disbursing reserve funds and securing a supplementary budget. To secure supplementary budgets, national bonds and loans were issued for restructuring funds for small-and-medium-sized enterprises (SME) [28]. In 2020, supplementary budgeting was done four times in a single year. It is a rare practice in national budgeting and has not been implemented since 1961 [29]. Regardless, the Korean National Assembly approved supplementary budgets, considering the urgency, necessity, and effectiveness of the COVID-19 response projects.

To respond to COVID-19, a supplementary budget of KRW 6.6 trillion (USD 5.6 billion), reserve funds of KRW 1.14 trillion (USD 0.97 billion), and KRW 364.6 billion (USD 309.61 million) through budget resolution transfers were secured by December 2020, resulting in a COVID-19 budget of a total of KRW 8.13 trillion (USD 6.90 billion) including the existing budget.

#### 3.2.2. Resource Mobilization and Allocation for COVID-19 Response

Although the prevention and treatment of infectious diseases were equally addressed, the priority of each varied at different times owing to the number of confirmed cases in the country and their economic effect. During the initial phase, the number of COVID-19 patients was small, and the focus was on the prevention of infection, such as border screening and epidemiological investigation, to prevent infected patients from entering Korea and contain the spread of the virus in the community [30]. However, after a large-scale outbreak, the focus shifted to securing beds to promptly treat severely ill patients and reduce fatalities [31]. Additionally, to sustain the healthcare delivery system and encourage healthcare institutions to participate in COVID-19 responses, healthcare institutions were provided with financial compensation and support.

Consequently, the highest public spending in 2020 was related to resilience (Table 1). A total of KRW 4461 billion (USD 3.78 billion) was spent on financial support for citizens, and KRW 1863 billion (USD 1.58 billion) on compensating healthcare institutions treating COVID-19 patients. The second highest budget spending was for detection. A total of KRW 844 billion (USD 0.72 billion) was spent on infection prevention and promotion, and KRW 338 billion (USD 0.29 billion) on border screening, diagnosis, and research. Self-quarantine and treatment costs were relatively small, at KRW 534 billion (USD 0.45 billion). These budgets are from the Korean Ministry of Health and Welfare (MoHW) and Coronavirus COVID-19 (KDCA). The actual expenditures in response to COVID-19 are estimated to be significantly higher. 

In response to COVID-19, a total of KRW 529.8 billion (USD 449.7 million) has been disbursed from the NHI fund (Table 2). COVID-19 billing by NHI for each main task [18,32].

#### 3.2.3. Purchasing and Financing Mechanism Adjustment during the Pandemic in Korea

To increase transparency in budget execution, the government, and public institutions utilize complex contract-payment processes [34]. However, several processes were simplified and relaxed during this unprecedented public health crisis to increase the speed of response. 

First, diagnostic testing typically requires 140 days to ascertain the diagnostic test agents. This period was reduced to 10 days for COVID-19-related diagnostic test agents during the pandemic; therefore, diagnostic testing could be performed on a large scale, which helped control and reduce the number of cases during the first outbreak. Second, after the first outbreak, the Korean government started a project to support healthcare institutions treating COVID-19 patients, by providing medical equipment to treat severely ill patients (e.g., ECMO and ventilators). However, the second outbreak occurred sooner than expected. Therefore, it was challenging to supply medical equipment in a timely manner with the existing contract process (open bidding), which lasted at least two months. Therefore, the government rushed to provide support through direct appointments with an active administrative committee. Third, generally, those insured under the NHI are required to pay a copayment of approximately 20–60% of the healthcare costs, to prevent moral hazards [35]. However, regarding COVID-19 related services, all copayments were exempted in consideration of the country’s accountability for the outbreak and the prevention of the further spread of the virus [24].

In the case of resilience, financial support systems were established to help medical institutions. The government was concerned that medical institutions would find it challenging to continue offering medical services if the prevalence was prolonged. The early reimbursement system, which pays 90% of the claimed medical fee before the review is completed by the Health Insurance Review and Assessment Service (HIRA), reduced the duration of the reimbursement process from 22 to 10 days. The advance reimbursement system was also introduced last year to pay 80% to 100% of medical fees, and adjust after a few months for medical institutions where the prevalence was severe. Additionally, generally, compensation for loss is principally paid after the losses are fixed; however, in the case of COVID-19, the government pays the approximated monthly compensation [36]. Moreover, the government temporarily lowered health insurance contributions to 30–50% in vulnerable groups experiencing a severe economic downfall owing to COVID-19, such as low-income groups and small business owners [37]. Therefore, there are typically few issues regarding healthcare inequity associated with different income levels [38].

## 4. Discussion

As of December 2020, Korea had a lower number of confirmed cases and fatality rate at a lower cost than OECD member countries [39]. In this regard, it is safe to conclude that Korea’s 3T strategy effectively responded to COVID-19 in the short term. The rapid mobilization of healthcare resources based on prepared laws, policies, and budgets contributed to this success [40,41,42]. Our study’s findings on Korea’s response to COVID-19 have the following implications.

A well-defined role between government departments is the first step to effectively respond to COVID-19 [43]. After the MERS outbreak in 2015, Korea regulated the infectious disease response system by prevalence volume in the Infectious Disease Control and Prevalence Act [44]. It specifies responsible organizations, resources, and finance mobilizations according to the risk level. Initially, the KCDA responded by using previously allocated resources. After the community spread, the Korean government established the Central Disaster and Safety Countermeasures Headquarters, controlled by the prime minister, to cooperate with all central and local governments. Therefore, the Korean government could secure the necessary resources for quarantine and treatment and avoid inefficiencies, such as duplicated allocations. This result emphasizes the importance of establishing specific responsibilities of organizations and government-wide cooperation [45].

The flexibility of health financing, which ensures reprioritizing spending, liquidity, and timely fund disbursement, can efficiently and effectively help countries respond to emergencies [46]. Flexible finance utilization was conducted as a prompt health reform based on the infectious disease response system established after the outbreak of the MERS [47]. Korea had an expenditure process that made decisions based on evidence for sustainable and transparent healthcare finance [48]. In principle, deliberation is conducted based on scientific evidence when listing items on the NHI or when purchasing medical supplies. However, because of the simplification of the COVID-19 process, the rapid expansion of the test and timely distribution were available. Accelerated procurement procedures are critical for a timely response to COVID-19 [49]. However, they should not compromise integrity. Therefore, the Korean government managed resources and budgets through the IT system to ensure transparency.

However, we found several reasons why Korea’s response is not sustainable in the long term. Using NHI finances may resolve inequality and further enhance the effectiveness of responses. The Korean government used NHI finances to provide testing and quarantine treatment for all suspected and confirmed COVID-19 patients [38]. Additionally, by reducing insurance premiums for the population whose income suddenly decreased owing to COVID-19, efforts were made to prevent unmet medical demand owing to the loss of eligibility. However, using NHI to respond to the pandemic may negatively affect national health insurance finances in the long run. NHI expenditures did not increase significantly in 2020. This was attributed to reduced medical use owing to the fear of infection during the COVID-19 pandemic [50,51]. 

The lack of public resources in the healthcare field exacerbates the challenges of resource mobilization and imposes unnecessary expenditure [52]. The budget for responding to infectious diseases and isolation beds was prepared before COVID-19; however, it was insufficient for this large-scale pandemic. It was difficult to mobilize private medical resources, and it was especially challenging to mobilize intensive care units and doctors capable of treating critically ill patients. To secure facilities and manpower, the government compensated for up to ten times the existing price for facilities and three times the existing wages for medical staff [53]. A total of KRW 895.8 billion (USD 760.8 million) was paid to compensate for losses at healthcare institutions after the first confirmed case in Korea (20 January 2020) until 31 January 2021 [18,32]. Nevertheless, it was challenging to mobilize medical staff and facilities immediately when the number of patients increased rapidly. This experience necessitates investment to reinforce the public sector [54,55].

The socioeconomic environment also suggests that a change in the COVID-19 response strategy is necessary for the long term. Korea implemented social distancing in its early stages as a strategy to reduce the number of confirmed cases [6]. As the COVID-19 outbreak lasted longer than expected, Korea was affected both socially and economically [39]. Socially, the polarization of education levels increased depression, and a care gap occurred [56,57,58]. Economically, the economic growth rate was lower than that in the previous year [39]. Accordingly, from 2021, Korea shifted its COVID-19 response goal from containment to daily recovery and focused on improving vaccination rates and reinforcing treatment capabilities [59]. Consequently, in 2021, the total number of confirmed COVID-19 cases was 570,095 and the death toll was 4663 [60]. Compared with 2020, the number of confirmed cases increased 9.4 times. However, the death rate decreased by 0.8 percentage points. These results show that the response priority should be selected in consideration of the epidemiologic characteristics of infectious diseases, vaccination rates, and socioeconomic effects.

This study has some limitations. First, the data used, and the document reviewed were from 2020, the first year of the COVID-19 pandemic. Moreover, Korea did relatively well in controlling the COVID-19. Updated and new data are necessary for future studies. Additionally, because we only used documents that were open to the public, it was difficult to precisely distinguish where the budget was used for detection and response. Despite these limitations, our study reviewed Korea’s strategies for the initial response to COVID-19 and the costs involved. Additionally, empirical cases that facilitated flexible and rapid financial utilization by relaxing regulations on securing and using budgets were presented. This is meaningful in that it can support policymakers in each country in making decisions about responding to infectious diseases.

## 5. Conclusions

Korea’s NHI and infectious disease response system, which was amended after the outbreak of the MERS, facilitated an initial response to the COVID-19 outbreak. Korea could provide timely quarantine and treatment services with a supplementary budget and administrative support secured during the response process. Consequently, it could maintain a low incidence and fatality rate compared with other countries at the time. However, our results suggest the need to enhance the financial stability of the NHI and the proportion of the public sector among health and medical resources for a sustainable and stable response to the COVID-19 pandemic.

## Figures and Tables

**Table 1:** COVID-19 response budget by category in 2020.

	Total	Common	Detection		Response	Resilience	
		Infection Prevention System	Border Screening, Diagnosis, and Research	Infection Prevention, Promotion	Self-Quarantine, Treatment	Support, Compensations	Livelihood Support for Citizens
Amount	8132 (USD 6.90 billion)	93 (USD 0.08 billion)	338 (USD 0.29 billion)	844 (USD 0.72 billion)	534 (USD 0.45 billion)	1863 (USD 1.58 billion)	4461 (USD 3.78 billion)
%	100.0	1.1	4.2	10.4	6.6	22.9	54.9

Unit: billion KRW, as of 1 December 2020. (1) Original budget: KRW 19,299,000,000 (at the end of January 2020). (2) Supplementary budgets: KRW 1,143,836,000,000 (1st–6th, including transferable funds); 5th (KDCA) KRW 4,560,000,000. (3) Supplementary budgeting. 1st (17 March 2020): KRW 3,667,540,000,000 (transferable funds KRW Δ35,628,000,000) = KRW 3,631,912,000,000; 3rd (3 July 2020): KRW 1,088,751,000,000; 4th (22 September 2020): KRW 1,668,376,000,000 (MoHW) + KRW 215,336,000,000 (KDCA) = KRW 1,883,712,000,000.

**Table 2:** COVID-19 billing from the National Health Insurance for each main task [33].

Category	Primary Task		Billing Amount (100 Million KRW)
Prevention of the occurrence and spread of COVID-19	① Infection prevention management for high-risk groups		818
	② Isolation room management or admission for new inpatients at nursing facilities/psychiatric hospitals		Nursing facilities 38 Psychiatric hospitals 3.2
Improvement of COVID-19 diagnostic testing	① COVID-19 PCR test–Single sample test		1803
	② COVID-19 PCR test–Pooled sample test		1st stage (group) 95 2nd stage (individual) 0.86
	③ Rapid PCR COVID-19 screening for emergency patients		19
	④ Integrated diagnostic PCR test for COVID-19 and influenza		4
	⑤ COVID-19 rapid antigen test		1.6
	Support for the treatment of severe COVID-19 patients	① Isolation room admissions	1245
		② Admission to intensive care units and management of negative pressure rooms in wards dedicated to treating severe patients	34
	Support for the treatment of mild COVID-19 patients	① Patient management at community treatment centers	84
		② Expansion of benefit criteria (increased maximum number to be reimbursed for treatment at home)	-
	Support for the treatment of emergency patients	① Isolated treatment areas at emergency treatment centers for severely ill patients	149
		② Emergency treatment management at screening centers	69
Support for new systems/institutions created for effective COVID-19 response	① National Safety Hospitals		603
	② Respiratory clinics		48
	② Medical fee for telemedicine		Telemedicine 218 Proxy prescription 64
	Reinforcing the treatment of patients experiencing difficulties owing to COVID-19	① Dialysis patients	Self-quarantine 0.78
		② COVID-19-related depression	Confirmed cases 0.25
	Expanding support for healthcare professionals and resolving issues at healthcare institutions	① Nighttime nursing care	0.2
		② Grace period for status reports regarding facilities and personnel	-
		③ COVID-19 patients are exempted from the comprehensive/new comprehensive fee systems	-
Total			Approximately 5298 (449.7 million USD)

## Data Availability

Not applicable.
